# Making and managing medical anomalies: Exploring the classification of ‘medically unexplained symptoms’

**DOI:** 10.1177/0306312720940405

**Published:** 2020-07-15

**Authors:** Erik Børve Rasmussen

**Affiliations:** 1 Oslo Metropolitan University, Norway

**Keywords:** ambiguity, anomalies, classification, medical science, medically unexplained symptoms

## Abstract

This article explores the making and management of anomaly in scientific work, taking ‘medically unexplained symptoms’ (MUS) as its case. MUS is a category used to characterize health conditions that are widely held to be ambiguous, in terms of their nature, causes and treatment. It has been suggested that MUS is a ‘wastebasket diagnosis’. However, although a powerful metaphor, it does neither the category nor the profession justice: Unlike waste in a wastebasket, unexplained symptoms are not discarded but *contained*, not ejected but *managed*. Rather than a ‘wastebasket’, I propose that we instead think about it as a ‘junk drawer’. A junk drawer is an ordering device whose function is the containment of things we want to keep but have nowhere else to put. Based on a critical document analysis of the research literature on MUS (107 research articles from 10 medical journals, published 2001–2016), the article explores how the MUS category is constituted and managed as a junk drawer in medical science.

## Introduction

In this article I explore the making and management of anomaly in scientific work, taking ‘medically unexplained symptoms’ (MUS) as my case. MUS is a category used to characterize health conditions widely held to be ambiguous in terms of their nature, causes and treatment (O’Leary, 2018). Sometimes referred to as ‘uncertain illness’ (Dumit, 2006), ‘illness without disease’ (Aarseth et al., 2016: 1391), ‘illness that cannot be diagnosed’ (Jutel, 2010: 230) or ‘symptoms that cannot be classified’ (Kornelsen et al., 2016: 367), MUS are known to cause a range of problems for doctors and their patients (Brown, 2007; Czachowski et al., 2011; Shattock et al., 2013). Patients with MUS are highly unpopular, as indicated by medicine’s use of unflattering monikers such as ‘frequent flyers’, ‘thick folder patients’ (Greco, 2012) and ‘heart-sink patients’ (Mathers and Gask, 1995), the latter so called because seeing the patients’ names on the appointment schedule is said to make a doctor’s heart sink (O’Dowd, 1988: 528). Although figures vary, it is generally agreed by researchers to be one of the largest categories of complaints in primary care (Brown, 2007; Greco, 2012; O’Leary, 2018).

The ambiguity of the clinic resonates with the ambiguity in medical science: As noted by members of the medical science community, research into MUS has been plagued by its lack of widely accepted modes of classification (Olde Hartman et al., 2008; Salmon, 2007: 247). The effects of this can be seen, for instance, in the confusingly unclear prevalence rates of MUS consultations in primary care, which have been estimated at 10–15% (Shattock et al., 2013) but also at 20% (Schaefert et al., 2013), 20–30% (Aiarzaguena et al., 2007), 3–39% (Koch et al., 2009) and 25–50% (Olde Hartman et al., 2004). The MUS category thus seemingly applies to anywhere between 3 and 50% of all primary care consultations. Moreover, years of research have not yielded any widely agreed evidence-based treatments, and in some cases (e.g. chronic fatigue syndrome, irritable bowel syndrome and multiple chemical sensitivity), the epistemic status of medical knowledge is loudly contested, often with patient activists and doctors pitted against each other (Aronowitz, 1998; Barker, 2010; Dumit, 2006; Lian and Nettleton, 2015).

In a review of the medical research literature, Jutel (2010) characterized the MUS category as ‘a wastebasket diagnosis’, a metaphor that has been taken up (e.g. Dimsdale et al., 2011). Wastebaskets are effective ordering devices, offering storage for discarded materials. However, although a powerful metaphor, it does neither the category nor the profession justice: Unlike waste in a wastebasket, unexplained symptoms are not discarded but *contained*, not ejected but *managed*. Just as patients with MUS are not chucked from the clinic, the category is not jettisoned from the jurisdiction of medical science. In fact, despite the lack of unitary classification, medically unexplained symptoms have become an increasingly hot topic of scientific inquiry. According to Web of Science, 951 research articles that topicalize MUS have been published in English between 1992 (earliest recorded) and 2018, with 5% (N = 47) published in the first 10 years compared to 73% (N = 692) in the last ten.^1^ Although the numbers are not necessarily entirely representative, they capture a real surge in scientific activity: Research is booming. Less is known, however, about how medical scientists, given the lack of consensus, actually go about classifying MUS, how the category is understood and used in scientific work. Learning how is the aim of the present article.

To that end, I propose another metaphor. Rather than a wastebasket, I suggest that we think about MUS as a ‘junk drawer’. As I use it here, a junk drawer is a concept, referring to a particular kind of ordering device whose function is the containment of things we want to keep but have nowhere else to put. It frees us from having to leave disorderly things lying about, or from having to put them into neatly ordered drawers where they do not belong. Its job, then, is to help *maintain order by containing disorder*. Whereas a wastebasket is for getting rid of disorder, a junk drawer is for storage. As such, the latter facilitates future attempts at reordering: for puzzles to be solved or ‘junk’ to be reappraised at a later juncture.^2^

In performing its ‘containment function’, a junk drawer also reveals ‘imperfections’ in the system. It does this in two ways. First, in its very establishment and use: A ‘junk drawer category’ is constructed to manage imperfections in the current cultural order that result from a lack of fit with material reality. Thus, the presence of a ‘junk drawer’ is an indication of the mismatch between reality and the classification system with which reality is grasped and wherein the category is embedded. It thus simultaneously expresses our imperfect grasp of reality and our pragmatic capacity to adapt – the more visible the category, the stronger the reminder. Second, systemic imperfections are revealed in the mundane experience with which we are confronted when opening the junk drawer to review the contents. Doing so reminds us that there is an outright mess in the middle of our system. As such, over time a junk drawer can signal the need to tidy, reconfigure and reorder. Omitting to inspect it, on the other hand, can make us forget the mess, deluding us with a pleasant sense of order and control.

In the following, I will provide a detailed examination of how medical scientists make and manage MUS as a ‘junk drawer category’. To that end, I have conducted a critical document analysis of the medical research literature (107 articles published between 2001 and 2016). My questions are: How is the MUS category constituted in research? And how do researchers manage it and its content?

## Medical classification

Medicine is replete with formalized classification systems or ‘diagnostic manuals’, notably WHO’s *International Classification of Diseases and Health Related Problems* (ICD) and the *International Classification of Primary Care* (ICPC), and the American Psychiatric Association’s *Diagnostic and Statistical Manual of Mental Disorders* (DSM). They provide a shared ‘terminological standard’, allowing for the commensurable codification of experience that renders ‘the world equivalent across cultures, time, and geography’ (Timmermans and Epstein, 2010: 69). Originally designed for statistical purposes, they are now increasingly integrated as a means of communication and coordination within professional work organizations and health insurance schemes and are used across the globe as a tool of accountability by states, hospital managers and other stakeholders (Bowker and Star, 2000; Harrison, 2009; Timmermans and Berg, 2003).

Although the MUS category is obviously medical, it is not a formal diagnosis, nor listed as a category of its own in any of the main diagnostic manuals. It *was* listed as a criterion for a cluster of diagnoses (somatoform disorders) in the fourth edition of DSM, but was intentionally excluded from the fifth edition, published in 2013 (see American Psychiatric Association, 2013; for criticism see Frances, 2013). Yet medically unexplained symptoms are continually and increasingly the object of clinical research. Paradoxically, then, despite being first partially then completely excluded from the formalized medical classification systems, the MUS category is increasingly enlisted in medical research and is thus steadily becoming a central medical category; it is pushed to the fringe yet drawn towards the centre of medical science. Therefore, rather than symptoms that defy classification (e.g. Kornelsen et al., 2016), MUS could be more appropriately described as subject to routine classification in scientific work. How do scientists do this work when there are no formal or widely accepted classification standards, and how they draw on their existing conventions and classifications in that regard?

## Ambiguity and science

I explore the classification of MUS as a case of the larger theme of anomaly in science. A relevant piece of literature in this regard is that of Kuhn (2012). Following Kuhn, phenomena are not anomalous (or ambiguous, deviant, disorderly, strange, etc.) on their own, but within the context of a specific paradigm (an ‘exemplar’ or an accepted ‘problem-solution’ – see Barnes, 1982: 17–19). That is, phenomena are anomalous because they are found to deviate from ‘paradigm-induced expectation’ (Kuhn, 2012: 53), from what reality is supposedly like. This way, knowledge is causally implicated in making phenomena anomalous. It implies a relational perspective, between knowledge and anomaly. When transposed to the case studied here, this suggests that the anomalous character of MUS is the effect of their lack of fit with conventional expectation. Thus, it is against the backdrop of some shared epistemic convention that these symptoms are anomalous.

Anomalies are common in science, and often they are simply ignored (Barnes, 1982; Kuhn, 2012). When an anomaly is recognized as a relevant phenomenon for research, scientists will typically attempt to uncover if the deviation is a result of faulty equipment or some other form of error in the research process (Barnes, 1982). If, after this, the anomaly lingers, different procedures may be applied (e.g. Douglas, 2003: 48–49). Based on a sociological reading of Lakatos (1976), Bloor (1978) distinguishes two such procedures, namely ‘monster-barring’ and ‘monster-adjustment’ (the term ‘monster’ referring to the problematic character of the anomaly).^3^ Monster-barring involves techniques for dismissing anomalies (Bloor, 1978: 253), either symbolically or physically. A wastebasket (whether physical or symbolic) is therefore a ‘monster-barring device’ to manage discarded items. Monster-adjustment involves techniques for reinterpreting or altering the anomaly, ensuring its fit with the established order (Bloor, 1978: 254). Some anomalies, however, resist attempts to resolve them and become intractable and annoying (Barnes, 1982; Hacking, 2012; Star, 1985). In these cases, anomalies may turn into crises, fostering changes to the paradigm that made the phenomena anomalous to begin with (Kuhn, 2012).

As I suggest below, MUS are continuously subject to monster-adjustment, yet the procedure has yet to succeed in removing the anomalous character of these symptoms. Learning more about the function of the MUS category may give us some clues about whether it represents a crisis in the making. In the following, I will show the ways scientists make and manage MUS as a ‘junk drawer’, beginning first by outlining the methods employed in this work.

## Methods and materials

I analyse a sample of research articles that centrally or peripherally topicalize MUS. The focus is on MUS not as a health problem, but as a category enlisted and investigated by medical research. As the meaning and character of categories are determined in the situated practice of applying them (Barnes, 1982; Bloor, 1997), studying the application of the MUS category is a good way to understand its meaning, use and overall function in medical science. Here, I have studied the traces of the category’s application in the inscriptions found in research articles. Although inscriptions differ from the act of inscribing them, there is much to learn from studying them. Given that research articles as documents (Prior, 2003) are enlisted in systematic reviews, textbooks and procedural and policy guidelines, and have a bearing on the definition of MUS as a health issue and thus the provision of attention and funds in clinical practice and medical research, they are a ‘strategic site’ (Merton, 1987) for the study of scientific classification.

On 26 January 2017, I used Web of Science to search for research articles in English published between 2001 and 2016 with the phrase ‘medically unexplained symptoms’ either in the title, abstract or keywords.^4^ 2001 has been suggested as the year where MUS went from being a descriptive term to being a research category proper (Nettleton, 2006). The search yielded 753 articles. I limited this initial list to publications in the ten medical journals that had published most frequently on MUS in the period. From these journals, I read the ten most cited articles in each, apart from one in which I read twenty because of its unusually high output. After excluding three articles that did not match the criteria, I had a manageable sample of 107 articles (Table 1). The citation-based sampling procedure is skewed towards earlier publications because they have had more time to amass citations. However, the procedure yields a number of publications from each year and makes it easier to spot systematic changes – if any – in classification practices over time (however, I found no systematic changes). The choice to sample articles from only 10 journals might have introduced some homogeneity to the sample.

**Table 1.** Sampled articles.

	Journal	Discipline	Country	Published (read)
*1*	*Journal of Psychosomatic Research*	Psychiatry	Netherlands	95 (20)
*2*	*Psychosomatic Medicine*	Psychiatry	US	28 (10)
*3*	*BMC Family Practice*	Community medicine	UK	21 (10)
*4*	*General Hospital Psychiatry*	Psychiatry	US	18 (10)
*5*	*Family Practice*	Community medicine	UK	17 (10)
*6*	*Psychosomatics*	Psychiatry	US	14 (9)
*7*	*British Journal of General Practice*	Community medicine	UK	12 (9)
*8*	*Psychotherapy and Psychosomatics*	Psychiatry	Switzerland	11 (9)
*9*	*Psychological Medicine*	Psychiatry	UK	11 (10)
*10*	*Patient Education and Counseling*	Community medicine	Netherlands	10 (10)

Judging by the publication rates in the journals sampled, the MUS category is used mostly in the context of psychiatric and primary care research (most frequently in the former) (177/60), though MUS is invoked in *psychosomatic* contexts more often than in psychiatry in general. Comparing the increase in publications that topicalize MUS with the total number of publications in the journals sampled shows that 2001 was indeed a pivotal year: While never rising above 1% of the total output before 2001, output increased from 1.3% (N = 11) to 7.4% (N = 88) of all publications between 2001 and 2016.

My analysis centres on definitions and operationalizations. Definitions were commonly found in the opening sections of articles, operationalizations in the methods sections. As both definitions and operationalizations are salient forms of classification in science, analysing them offers important clues about the classification of MUS in scientific work.

Articulations of definitions and operationalizations may deviate from what researchers actually think and do in practice. For the purposes of this study, it is more important what researchers want to communicate than what they think for themselves. Deviations from actual practice are, however, a possible problem. In the analysis, I therefore focus on what seem to be straightforward differences in classification practices (e.g. it seems clear that researchers requiring four unexplained symptoms for admission in a study have classified differently from those requiring only one, even though we do not know how they actually counted). In the discussion, I raise the issue of how much crucial information is missing from the accounts.

I coded articulations of MUS thematically, first in a broad-brushed manner in Nvivo, then in a more finely grained manner in Word. During the analysis, I wrote memos subsequently incorporated in the analysis. I was interested in classification practices from the outset, but the interest in methodological variations *as* varied classification practices stemmed from engaging with the data. Early versions of the analysis have been presented to audiences of social scientists and medical researchers on four occasions.

All documents in the sample are listed in the *Appendix*, each with its own code consisting of two numbers (e.g. **1-3** or **5-7**). Thus, ‘**1-3**’ refers to the third most cited article in journal one, whereas ‘**5-7**’ refers to the seventh most cited article in journal five. When referring to documents from the sample in the analysis, I use this coding system.

## Constituting the junk drawer

### The core criterion

As an ordering device, a junk drawer is a means of controlling anomaly by containing it. The MUS category is thus a means to manage cases that are in some way considered anomalous. But what makes them anomalous? What defines a case of MUS? According to medical science, the most fundamental feature of MUS is the co-occurrence of present somatic symptoms and the absence of evidence/signs of somatic disease: Patients complain that something is physically wrong with them, but doctors find nothing to support that claim in or on their bodies. This is exemplified in the following list of brief and more or less explicit articulations, where MUS are defined as referring to:

**Example A**: ‘… any current principal somatic complaint reported by patients for which no definite medical diagnosis could be found by physical examination and appropriate investigation.’ (**1-1**, i.e. journal 1-article 1, 2001: 362)

**B**: ‘… patient-reported physical symptoms for which physicians cannot find corresponding physical pathology or for which the underlying physical pathology does not adequately account for the patient’s description of symptom severity or disability’ (**5-9**, 2010: 487)

**C**: ‘… patients’ experience of physical symptoms [that are] discordant with the degree of tissue abnormality found on objective tests or with other observable signs of illness.’ (**6-4**, 2002: 206)

**D**: ‘While most people experience at least some physical symptoms, a number of patients repeatedly attend with symptoms for which a conventional pathology cannot be identified.’ (**7-2**, 2003: 231).

**E**: ‘… persistent and distressing somatic symptoms for which adequate somatic examination does not reveal sufficient explanatory peripheral organ pathology.’ (**8-6**, 2012: 106-7)

**F**: ‘… physical symptoms without any sufficient organic findings’ (**10-4**, 2009: 207)

There are differences between the examples, such as the degree to which symptoms are unexplained – ranging from when examination does not yield ‘*sufficient* organic findings’ (**F**) or ‘pathology does not *adequately* account for’ the symptoms (**B**), to when ‘conventional pathology *cannot be identified*’ (**D**) or ‘physicians *cannot find* corresponding physical pathology’ (**B**) – or in the way in which some suggest a more limited membership (e.g. **E**: ‘*persistent* and *distressing* somatic symptoms’), whereas others are more inclusive (e.g. **A**: ‘*any* current principal somatic complaint’). Yet each example exhibits the same co-occurrence of present somatic symptoms and absent somatic signs of disease. This co-occurrence is the core of how MUS is defined in the vast majority of cases in my sample, regardless of the source journal, disciplinary identity, method or aims. In fact, only one articulation explicitly deviated from this convention (the definition included ‘the absence of identifiable organic pathology’ but the present symptom could be both ‘physical *and mental’* [**5-5**, 2004: 199]).

In medicine, the interest in this co-occurrence of present symptoms and missing signs seems self-evident. It is almost never discussed or thematized explicitly in the literature or in my sample (but see **2-10**). It may seem equally obvious to outsiders. However, for analytical purposes, we should estrange ourselves and ask what it is about the co-occurrence of present symptoms and absent signs that motivates the invocation of a ‘junk drawer category’. Why is it problematic to have the one but not the other? The answer has to do with epistemic convention. The co-occurrence is expressive of a discrepancy, an indication that somatic signs are expected to accompany somatic symptoms. In particular, the uses of ‘discordant with’ (**C**) and ‘without’ (**F**) indicate that an expectation is being violated: there should be concordance between ‘physical symptoms’ and ‘the degree of tissue abnormality’; ‘*sufficient* organic findings’ should accompany organic symptoms. It makes sense to point out that you have the one (somatic symptom) but not the other (somatic sign) if you expected to have both. From the point of view of actors expecting both, MUS thus make manifest feelings of ambiguity, of uncertainty, doubt and risk. MUS thus become problematic.

In some cases, MUS are not defined explicitly but simply referred to as a familiar concept whose meaning is taken for granted (e.g. **9-4**; **9-6**). Yet even in these cases of non-definitional articulation, MUS were clearly revealed in the methods sections of articles as being premised on the co-occurrence of present symptoms and absent signs. For instance, in **9-4**, the authors simply introduce MUS as symptoms that ‘occur frequently in all medical settings, and are associated with psychiatric disorder and reduced functioning’ (**9-4**, 2003: 519). But although the category is not initially defined, the core criterion of co-occurrence is revealed in the account of the operationalization procedures (my emphasis below):

Criteria for a medically unexplained episode …: (a) the patient presented with *physical symptoms*; (b) they received investigations for these; *and* (c) *the investigations and clinical examination revealed no abnormality*, or abnormalities that were thought to be trivial or incidental (**9-4**, 2003: 520).

Therefore, MUS are constituted as anomalies by their lack of fit with some ruling epistemic convention or paradigm (Barnes, 1982; Kuhn, 2012). As indicated both by the expectation that somatic symptoms should be accompanied (and indeed caused) by underlying somatic disease, and by the emphasis on terms such as ‘disease’, ‘organic’, ‘pathology’ and ‘tissue abnormality’, the paradigm in question is that of scientific biomedicine and its biomedical model of disease. Central to that model are the notions that: (i) psyche and soma (body) are separate domains (though not necessarily independent), (ii) symptoms are effects that should have causes, (iii) somatic symptoms should have somatic causes, known as ‘disease entities’, (iv) such entities may be detected upon physical examination (blood tests, imaging technologies, palpation, etc.) in the form of objective ‘signs of disease’ (tissue abnormalities, organic pathology, etc.), and, (v) upon detection, the objective signs explain the subjective symptom (e.g. Lock and Gordon, 1988). As historians of medicine and science have shown, this is a culturally contingent understanding of disease, contrasting sharply with, for instance, the symptom-oriented 17th century classification of Sydenham or the ‘humoral pathology’ of Galenic medicine (Jewson, 1976; Jutel, 2010; Porter, 1999). Likewise, the distinction between ‘objective signs’ and ‘subjective symptoms’ is a sociocultural achievement consisting of the symbolic decoupling of facts from their observer (Shapin and Schaffer, 2011). The biomedical paradigm is, however, an important influence, not least in the medical research into MUS: It is what makes the core criterion reasonable.

MUS may thus be characterized as anomalies constituted by their lack of fit with scientific biomedicine, by their violation of expectations induced by the biomedical paradigm. When MUS are defined by the co-occurrence of present symptoms and absent signs, therefore, it is not simply that signs, like a great number of things, *are not there*. Rather, from the point of view of biomedicine, they are *missing*. When they are missing, the symptom is unexplained, since underlying disease entities are the explanans, or what ‘does the explaining’ (i.e. causes), in biomedicine; or the symptom is dubious, since objective evidence are what identifies symptoms as real. It is thus against the background of these conventions that MUS are anomalous: their violation summons up the possibility that there is something medicine has not found or understood. The ‘discordance’ between what the patient says and what the doctor can find must thus be accounted for.

Another way of putting this is that the system of medical knowledge is causally implicated in the construction of MUS as a ‘junk drawer category’ – it causes the need for a category to manage anomalous symptoms. Cases of MUS thus become interestingly similar within the context of modern biomedicine, giving the category some sense of coherence. Typically, however, due to the framing of the core criterion, the deeper connection between biomedicine and the ambiguity of MUS is hidden from view.

### Framing the core criterion

We can discern two main ways of framing the core criterion, which I call the doxic and the heterodoxic framing. They form a continuum rather than a neat dichotomy, with some articulations ambiguously poised in the middle.

The doxic framing earns its name from its frequent occurrence in the sample and from the way it takes biomedicine for granted (doxa) as the basis for thinking about MUS. Its main characteristic is thus the silencing of how taking biomedicine for granted conceals its constitutive role in making MUS anomalous. The doxic framing thus centres on symptoms and patients, while keeping doctors and their knowledge more or less out of the picture. Consider the following example (an extended version of example **F** above, my emphasis):

International studies show that 10–20% of *patients* in primary care *suffer from physical symptoms without any sufficient organic findings*. In some medical specialties, such as gynaecology, neurology, or gastroenterology, in 30–70% of cases *no organic causes for the patients’ symptoms can be found. These patients* with medically unexplained symptoms (MUS) constitute an economically relevant group in the health care system, since they *receive many elaborate diagnostic examinations and medical interventions, in spite of the absence of an organic disease*. (**10-4**, 2009: 207)

The focus is clearly on symptoms and patients (and budgets). Of course, such a focus is not out of place in a medical context. But the (unintended) consequence that stems from this focus is important: By the very act of fixing its gaze on symptoms and patients, the text effectively turns its back on medical knowledge and medical professionals, rendering them almost invisible. Note, for instance, that we are told nothing of the doctors who are unable to come up with the right sort of evidence and the specific and limited techniques they have at their disposal in that regard, or that the demand for evidence of specific sorts results from the paradigm the doctors employ. Instead, the reader is confronted with *symptoms* that are ‘without … findings’ or for which ‘no organic causes … can be found’, and with expensive *patients* who ‘receive many elaborate diagnostic examinations’ (omitting the doctors who are providing them). The narrative presents MUS as having to do with symptoms and their patients, but not with the medical profession and its knowledge base that play a constitutive role in making these cases into medical problems in the first place. Doxa thus creates a problem, while the doxic framing deletes any trace of its involvement.

Accordingly, MUS seems like a quality inherent in the symptoms rather than an ascribed attribute resulting from the mismatch between those symptoms and the biomedical paradigm. The examples above (**A**–**F**) all frame MUS in this doxic way (other cases are less explicit, but more often than not, articulations take this form). The taken for granted character of the articulations of MUS also extends to concepts such as ‘medically explained’, ‘disease’, ‘pathology’, ‘objective’ and so forth – the symptoms *just are* subjective, just as the missing forms of evidence *just are* objective.

A minority of articles deviates from the doxic norm and instead employs what I have dubbed a heterodoxic framing. Most of these are by Peter Salmon, either alone (**10-1**) or in collaboration (e.g. **4-6**), underscoring the doxa of the doxic framing, so to speak. The heterodoxic framing rightfully earns its name from its rebellion against doxa. Within this frame, MUS are not a quality of the symptoms and the patients, but of the consulting clinician and her or his medical knowledge. Consider, for instance, the following articulations (my emphases):

**G**: ‘Many patients present to their general practitioner (GP) with *symptoms that the doctor thinks are medically unexplained* (MUSs)’ (**4-6**, 2007: 454).

**H**: ‘About 10–20% of patients present physical *symptoms* in primary care *that their general practitioners* (GPs) *believe are not explained by physical disease*’ (**4-8**, 2008: 104).

Compared to the examples of doxic framing above (**A**–**F**), the difference is syntactically negligible but semantically substantial: MUS are now a phenomenon that refers to *what doctors think* (**G**) or *believe* (**H**) about symptoms. In other words, the heterodoxic frame topicalizes medical knowledge and expert judgement as part of the nature of MUS. This radically changes the meaning of MUS, moving their referent from the ontological to the epistemic realm. On rare occasions, the heterodoxic framing is even more explicit and critical. For instance, **2-10** present MUS as a convenient name for ‘symptoms that elude diagnosis … even though it is a failure to understand them rather than the symptoms themselves that define [sic] them as “unexplained”’ (2006: 269). The lack of correlate ‘tissue abnormality’ for ‘physical symptoms’ (**C**) is no longer a story of the symptoms themselves, but of the physicians’ lack of understanding and of current imperfections in their knowledge. The MUS category expresses ‘a failure to understand’ symptoms, rather than anything in particular about those symptoms.

Thus, although the doxic and heterodoxic framings both make MUS about the co-occurrence of present somatic symptoms and absent somatic signs, they disagree on the implications: the heterodoxic framing foregrounds biomedicine and its practitioners, thus making the doctors’ knowledge and practices figure as important factors. It does not, however, fully recognize the causal role of biomedicine in making MUS anomalous: What is highlighted is the ignorance and impotence of practitioners, but nothing is said about the causal role of their positive knowledge, for example, how the biomedical paradigm makes it problematic to have somatic symptoms but not somatic signs of disease. It is never pointed out how it is what is known (knowledge), as much as what is not (ignorance), that makes MUS ambiguous and problematic. Thus, the constitutive dynamic between symptom and epistemic order is made obscure by both framings.

### Solid core, fuzzy boundaries

Apart from variations in framing, the sample is homogenous in its implicit agreement about the core criterion. But there are also important definitional variations, especially in the practices of establishing similarity between MUS and two other salient categories. The variations are interesting because they dramatically alter the scope and meaning of MUS, and thus about its constitution as a junk drawer in the epistemic order.

The first is ‘somatoform disorders’ (SD), referring to various forms of ‘somatization’, something a patient may be said to suffer from if (s)he is prone to ‘somatize’ (or ‘make somatic’), meaning that (s)he is likely to interpret and indeed experience mental illness as though it were bodily in kind. To somatize is to *attribute* pain to the body that is actually in the mind (e.g. Greco, 2012; Jutel, 2010). As stated above, SD were organized as a cluster of psychiatric diagnoses in the *Diagnostic and Statistical Manual of Mental Disorders* until its fifth revision in 2013 – but the practice of connecting SD with MUS has not stopped (e.g. **3-9**).

MUS connect with SD in one of two ways. Some present SD as a special and more serious type of MUS. For instance, in **1-13** (2006: 169): ‘Somatization may be viewed as a phenomenon characterized by clinically significant yet medically unexplained symptoms (MUS), causing distress, disability, or maladaptive behaviour.’ An SD is thus a ‘clinically significant’ MUS, meaning that other MUS are more trivial or a ‘low threshold variant’ of SD (**2-1**, 2007: 882). Others have articulated the connection schematically, presenting ‘somatizers’ as ‘people with *both* medically unexplained symptoms *and* anxiety and depression’ (**7-5** 2011: e295). The important thing to note is how MUS are articulated as a wide category that include SD but also less distressing or disabling symptoms. In other cases, MUS were articulated as synonymous with SD, such as in the following: ‘Somatoform (i.e. medically unexplained) symptoms are common in the general population’ (**4-3**, 2008: 349). Synonymous articulation also occurs when SD are defined exactly like MUS, as ‘the presence of somatic symptoms that cannot be sufficiently explained by organic etiology’ (**2-3**, 2008: 716). Sometimes articulation simply glides from one concept to the other as though it made no difference. For instance, in **3-3** (2009: 2), articulations transition seamlessly from ‘the field of somatisation’ to ‘managing MUS’ and ‘MUS patients’. Such transitioning is sensible if one accepts that MUS and SD are synonymous, but senseless if one disagrees (as apparently do **1-13**, **2-1** and **7-5** above).

The connection with SD is a frequent occurrence in the sample and in many cases the documents are as much about SD as they are about MUS. This is likely because most SD diagnoses have MUS as a core criterion. The explicit association with SD was stronger in articles in psychiatric than community medicine journals (~55% vs. ~15%): SD is a psychiatric category.

The second category with which MUS are frequently connected is ‘functional somatic syndromes’ (FSS). FSS refers to clusters of symptoms that are considered well-described and non-trivial complaints. FSS are typically exemplified by diagnoses such as chronic fatigue syndrome, fibromyalgia and irritable bowel syndrome, which, incidentally, are the most common exemplars of MUS in social science.^5^ As with SD, FSS were articulated either as a more serious version of MUS, or as synonymous with them. For instance, consider the following examples (my emphasis):

**I**: ‘Many studies on unexplained physical symptoms have been performed among patients with diagnosed chronic *functional syndromes* such as fibromyalgia, irritable bowel syndrome, or chronic fatigue syndrome …. However, *these syndromes represent the far end of the spectrum* of unexplained symptoms.’ (**1-12** 2008: 265).

**J**: ‘*Medically unexplained syndromes, also known as functional somatic syndromes (FSS)*, are defined as ‘syndromes characterized more by symptoms, suffering and disability than by consistently demonstrable tissue abnormality’ (Barsky & Borus, 1999, p. 910). They are found in every medical speciality and include syndromes such as fibromyalgia, chronic fatigue syndrome (CFS), and irritable bowel syndrome (IBS).’ (**9-3**, 2005 p. 583)

Whereas **I** treats FSS as a special case of MUS (‘the far end of the spectrum’), **J** treats MUS and FSS as synonymous (‘also known as’). Note also the overlapping examples (chronic fatigue syndrome, fibromyalgia and irritable bowel syndrome): The authors describe the same conditions but disagree whether they are special or normal cases of MUS. **I** and **J** are not unique – as seen in, for instance, **1-3** (2001: 549) and **4-9** (2010: 2) respectively.

For our purposes, the connections with SD and FSS are interesting because they variously alter the scope and meaning of the MUS category, thus indicating differing practical applications. Moreover, they indicate differing thresholds for declaring something an anomaly. For instance, since FSS are relatively ordered and non-trivial conditions, articulating MUS as *synonymous* with FSS (as in **J**) makes it a smaller, more ordered and severe category than if FSS made up only part of the MUS spectrum (as in **I**). The same is true of SD but the connection with SD is interesting for an additional reason: If the physical symptom can be reframed as a mental symptom that the patient has wrongly attributed to the body, the ambiguity of present somatic symptoms and absent somatic signs dissipates (see also Jutel, 2010). Thus, attempting to connect MUS with SD could be construed as a case of ‘monster adjustment’ (Bloor, 1978, more below): If widely accepted, it will effectively remove the anomalous character of MUS. Due to the aforementioned revisions to medical classification, however, that is an unlikely scenario.

To sum up the analysis so far: The core criterion of MUS is the co-occurrence of present somatic symptoms and absent somatic signs. This criterion implicates the biomedical paradigm as a constituent factor. In the vast majority of cases, the category is framed in a way that conceals this from the reader. Thus, the core criterion comes across as capturing an important feature of a patient group, a group that can therefore meaningfully be classified as one. It is unclear, however, if the MUS category applies to all cases that fulfil this criterion or is limited to a subset. This implies an ambiguity of meaning. In the next part, we can see how this ambiguity extends to practical applications in research.

## Managing the junk drawer

A junk drawer is for containing disorder, for anomalous cases. What characterizes medical scientists’ management of the junk drawer? That is, how is the MUS category *operationalized* in research? The answer is that operationalization varies tremendously, leading to the drawer being filled with the experiences and characteristics of very different groups of patients. Though one might have expected classification to vary according to disciplinary boundaries (between community medicine and psychiatry), my analysis reveals that, in the main, it does not. Here I present the most important ways in which operationalizing practices varied.

### Operationalizing the core criterion

Among those that articulate an interpretation of the core criterion (which many do not, e.g. **10-7**; **10-8**),^6^ the key difference is in how they draw the line between MUS and medically explained symptoms (MES), e.g. **1-15**) and in how they manage the ambiguous ‘grey area’ between the clearly unexplained and the clearly explained. For our purposes, it is intriguing how the studies variously include or exclude patients from consideration, and thus enables or prevents their illness experience from becoming part of what ‘MUS-knowledge’ is knowledge about. The grey area thus consists of the cases that are either recognized as anomalies or ignored. The grey area can, moreover, be of considerable size, as doctors widely disagree whether a symptom is (fully) explained (see **1-13**; **3-9**; **5-9**). Thus, the difference between an inclusive or a restrictive operationalization of the core criterion decides which patients that are included in scientific research into MUS. It tells us of the function(s) of the junk drawer in science.

Some researchers operationalize MUS inclusively as containing all symptoms not fully explained by ‘tissue abnormalities’ or other signs, including cases of doubt. If not MES, then MUS. For instance, in one study, symptoms were classified ‘as either “medically explained” or “(partly) medically unexplained”’ (**1-15**, 2011: 144). Likewise, in another study (**9-4**, 2003: 520), both ‘definitely’ and ‘probably unexplained’ symptoms were treated as MUS. Thus, the grey area is incorporated into MUS. This is reminiscent of the definitions we saw above, where MUS include cases where ‘pathology does not *adequately* account for’ the symptom (**B**) or where there are not ‘*sufficient* organic findings’ (**F**). There may be pathology, and there may be organic findings, but not to a satisfactory degree. In some such cases (e.g. **2-9**; **8-3**), the grey area was operationalized as a subcategory of MUS called ‘minor acute illness’ (MAI), differentiated from ‘somatization’ (severe MUS). Although more finely grained, the procedure is just as inclusive (cases of MAI were included).

Others operationalize MUS restrictively as limited to cases that are fully unexplained, meaning that cases of doubt were excluded from the category. For instance, in one study, ‘where there was uncertainty, the case was reviewed by two other raters [other than the one who was uncertain] … and categorized as MUS only if agreement was unanimous’ (**5-8**, 2010: 480). Doubt is thus either resolved or kept clear of the MUS category. This latter strategy of removing doubt (i.e. disambiguation) is rigged in different ways. For instance, in **4-2**, two researchers classified each case as MUS or MES, and in cases of disagreement they would discuss the matter until agreement was reached. The grey area is thus distributed between MUS and MES, thereby maintaining an orderly dichotomy. Compare this with **6-3**, where the goal was instead to ensure the integrity of MUS and MES, keeping ambiguity away from both:

Symptoms that could be attributed to a known medical condition (i.e., known somatic disease or pathophysiologic dysfunction) were regarded as ‘medically explained.’ In cases where no pathologic findings could be detected, symptoms were regarded as ‘medically unexplained.’ If the findings were ambiguous, symptoms were regarded as ‘mixed.’ (**6-3**, 2011: 265-6)

Cases of doubt are categorized as ‘mixed’ and thus kept out of the MUS category. A similar strategy was devised in **4-9**, where cases were classified ‘as either presenting with (1) well-defined physical disease, (2) probably well-defined physical disease, (3) MUS, (4) mental disorder with connected physical symptoms or (5) no physical problem’ (2010: 2), and only categories (1) and (3) were included in the study.

Notably, a few studies are not interested in what the patients *have* per se but in what doctors (and sometimes patients themselves) *believe* they have (e.g. **1-7**; **4-4**; **4-6**; **10-2**). For instance, in **1-7** (2005: 256), MUS was operationalized using ‘a procedure … to identify patients that, *in the doctor’s opinion*, have unexplained symptoms’. This manner of operationalization is typically found with the heterodoxic framing discussed above (though not always, e.g. **6-2**). But whereas the semantic difference between doxic and heterodoxic framings is dramatic, the operational differences are more superficial: whether recognized or not, the bottom line is always a doctor’s professional belief that, following physical examination, a case is unexplained by physical disease. There are certainly procedural differences to ensure the validity of the verdict but it is always ‘possible that some symptoms identified as “unexplained” might prove to have a pathological cause’ (**1-7**, 2005: 256). Accordingly, the manner in which the grey area is dealt with in these cases varies along the same dimensions, as outlined above.

### Additional criteria

In addition to the core criterion, studies have often enlisted additional criteria. The four most common types are symptom *count, impact* and *persistence* and the *frequency of attendance* at the clinic (or some other measure of health care use). The first three characterize the complexity and severity of the complaint: Count specifies a minimum number of symptoms, persistence specifies a minimum timespan the symptom must have existed, and impact specifies the power of the symptom, typically to distance MUS from ‘trivia’. So for instance, in some cases, research includes patients with single, recently onset and merely bothersome symptoms, whereas in other cases it is limited to patients with multiple and seriously debilitating symptoms lasting six months or more. Frequency of attendance is included either because repeatedly seeking help is considered a sign that the patient has MUS, a form of behaviour revealing that the patient is ‘excessively’ worried (**3-5**; **6-2**), or to ensure that the patient group in question consists of ‘high service users’ (**3-5**), thus delimiting MUS from an ‘economically irrelevant’ group of patients who do not suffer (enough), or who do suffer but for various reasons choose not to seek professional support (similar remarks in Jutel, 2010: 236). The two are not mutually exclusive, but the former is strongly associated with somatization studies and studies into the predictive effects of ‘illness perceptions’ (e.g. **1-6**; **2-5**). The latter is rarely articulated but is indicated by frequent reference to MUS as an expensive, resource-demanding group (e.g. **10-4**, 2009: 207).

Based on the various uses of these additional criteria, we can distinguish between more or less inclusive classifications and specify the types of conditions and patients that are variously omitted or included in the junk drawer.

First, we can note that some studies (e.g. **4-7**; **4-10**; **7-9**; **9-4**) feature *none* of the criteria, meaning that ‘everything and anything’ passes for MUS, as long as there are no – or insufficient – findings. Thus, patients can have one symptom or many, the symptoms may have started recently or lingered for years, they may be trivial or seriously disabling functional impairments and the patients may be strangers or regulars at the clinic.

More often than not, however, one or more additional criteria are enlisted. For instance, in a study of predictors of psychiatric morbidity, impact was a criterion (though not a very specific one): ‘*Mild symptoms* that had not led to consultation with a health care professional … or to marked interference with normal activities *were excluded’* (**1-19**, 2006: 126). Another study combined impact with persistence criteria, instructing ‘The GPs at each general practice [to select] patients with *serious persistent* medically unexplained symptoms’ (**8-1**, 2006: 283). Other studies, such as the following study measuring the effects of acupuncture on MUS patients, used a more extensive and elaborate set of criteria (my emphases):

‘Criteria for ‘persistent medically unexplained physical symptoms’

a. The presentation of a physical symptom

b. The symptom had existed for *at least 3 months*

c. It had caused clinically *significant distress or impairment*

d. It could not be explained by physical disease, that is; ‘*physical symptoms for which no clear or consistent organic pathology can be demonstrated’*

Other inclusion criteria (from electronic record search) Had consulted GPs (clinic, telephone or home consultations) *8 or more times in previous 12 months’* (**7-5**, 2011: e296)

We can recognize the operationalized core criterion in item ‘d’ and we can see that frequency of attendance (‘8 or more times in previous 12 months’), persistence (‘at least three months’) and impact (‘clinically significant distress or impairment’) are listed. Symptom count, however, was not relevant to inclusion. Some studies used the same set of criteria but operationalized them differently. For instance, in a study testing the effectiveness of mindfulness-based cognitive therapy, (**8-8**, 2013: 300), the frequency of attendance was operationalized as relative (‘the 10% most frequently attending male and female patients’) rather than absolute ( more than eight consultations per year), the persistence criterion was stricter (‘at least six months’ rather than ‘at least 3’) and the impact criterion was more lax (‘experiencing functional impairment’ vs. ‘significant distress or impairment’). With regards to which patients and which health problems were included in research about MUS, the difference between **7-5** and **8-8** is substantial, just as the difference between these studies and those that enlisted no additional criteria (e.g. **7-9** above) is enormous.

To compare the examples reviewed so far, some studies make high and gender-specific demands on symptom count. For instance, a study testing the validity of a diagnostic instrument^7^ operationalized MUS using the criteria for ‘abridged somatization’ – a diagnosis that requires ‘a history of six medically unexplained symptoms for women and four medically unexplained symptoms for men’ (**6-1**, 2006: 393).^8^ Inclusion also required symptoms that were ‘significantly distressing’ (p. 393) but the persistence and frequency of attendance were not specified. Other studies (e.g. **6-2** and **6-6**) made the same gender sensitive demand on symptom count, but with different criteria added. For instance, a study evaluating a psychotherapeutic intervention added criteria for persistence (‘persistent symptoms for at least three months’) and frequency of attendance (‘5 or more annual doctor’s visits or 2 hospitalizations during the last year as a result of the respective symptoms’) and used very specific if broadly inclusive means to determine impact (**8-7**, 2007: 340).^9^ The reason for demanding a higher symptom count for women in these cases is to account for the fact that on average women report more symptoms than men do. The idea is, presumably, that this discrepancy has to do with gendered illness behaviour and illness experience more than with the actual conditions ‘themselves’. Researchers thus use gendered criteria to compensate for differences in male/female illness behaviour and illness experience.^10^

Other studies demanded multiple symptoms but typically set the bar at two or more without making gender specific demands (e.g. **6-5** and **8-4**). The difference is far from trivial: whereas, say, **8-4** includes women with four and five severe and persistent symptoms, **6-1** does not. Likewise, the difference between **6-1** and **8-7** is substantial: for instance, the latter discounts male patients with five severe and persistent symptoms if they have sought help only four times in the last year; the former does not. And the comparative difference between studies demanding multiple symptoms (e.g. **6-1** or **8-7**) and those that do not (e.g. **4-7** or **8-1**) is enormous. For instance, in one study, GPs classified 33% of the patient population in primary care as having MUS, but only about 3% as having multiple MUS (**3-9**, 2014: 3).

### Unwitting variations

As is apparent from the examples reviewed, the MUS category is used to study health problems that are potentially highly dissimilar in medically relevant ways. Thus, researchers cluster and store information about patients whose needs are potentially very different in the same category. These patterned variations help explain the extraordinarily confused prevalence estimates of MUS reported by medical scientists (3–50% of all primary care consultations, see introduction). Yet the variations take on extra significance because the medical scientists are strangely silent about them. During a close reading of the research literature, this absence is striking: there are almost no intertextual references or discussions in the sample to how others have classified MUS and how one’s own approach differs. Thus, medical scientists seem *unaware* of the tremendous variations in their management of the MUS category.

It is not uncommon to find researchers operating with differing criteria, but when methodological variations are accompanied by silence and widespread unawareness, it can be problematic. For clinicians who are expected more and more to keep up with the latest research, it can lead to confusion and exacerbation of ‘research-based uncertainty’ (Timmermans and Angell, 2001). It also seemingly causes confusion among medical scientists themselves, who uncritically draw on conclusions from studies that have operationalized MUS very differently from themselves (different criteria, data, procedures, etc.). For instance, **7-4** estimates the prevalence of MUS in primary care based on insights from two studies: One is a study of somatization disorders and ‘disorders of the mood’ (Bridges and Goldberg, 1985: 536) which is not how **7-4** defines or operationalizes MUS; the other studies patients with ‘common symptoms’ at ambulatory outpatient clinics (Kroenke and Mangelsdorff, 1989), which is not primary care. Other examples abound. To the extent that MUS was an ambiguous category to begin with, medical scientists’ management of the category seems to produce more ambiguity rather than less.

## Discussion and concluding remarks

I have omitted some details concerning operationalization – notably the places (e.g. primary or secondary care, Manchester or Munich) and players (assistants, consulting physicians, patients, etc.) involved, and the varied uses of classification instruments and (more rarely) selected codes from WHO’s main diagnostic manuals used to operationalize MUS (see **3-6**; **4-3**; **5-3**; and **8-7** for examples). Moreover, I have given scant attention to what researchers hope to achieve by enlisting the MUS category in their work. Most of the articles sampled aim either at improving the quality of care for patients or the working conditions of doctors by testing various interventions or assessing or improving the classification of MUS or related complaints, often by testing novel categories and techniques for lumping together and splitting patient groups into categories. Among the latter, many express critical views of the MUS category and aim to avoid or replace it with other classifications.^11^ As pointed out by Greco (2012), these critical-constructive projects express a motivation to improve care for patients and the working conditions of clinicians and can be read as an attempt to soften the tension between patients and professionals that MUS are associated with. Here, however, my focus has been on how the category is constructed and applied in science and what the effects might be of that – regardless of the researchers’ aims.

The analysis has demonstrated how MUS is constituted as a ‘junk drawer category’, and how this category is managed in medical research. First, I have shown that the core criterion of MUS as a category is the co-occurrence of present somatic symptoms and absent somatic signs of disease, and that, fundamentally, this criterion is an expression that MUS violate expectations induced by the biomedical paradigm. Biomedicine as epistemic convention is therefore causally implicated in making symptoms anomalous and this ‘paradigm-induced’ anomalous character is what constitutes MUS as a category. In other words, the junk drawer is constituted as a contravention of the existing order. The majority of articles, however, hide the constitutive role of biomedicine, making the core criterion an ontological fact about the symptoms, rather than an epistemic fact about the beliefs and practices of the profession. Thus, the nature of the MUS category as a junk drawer is concealed.

Second, I have shown patterns of substantial variation in how the category is managed, focusing on the most important variations in what scientists think belongs in the MUS category. Thus, the analysis reveals a lack of consensus in the proper functioning of the junk drawer. I interpret this lack of consensus as a disagreement about the threshold where anomalous symptoms become important for science. On the one end are those who manage the category restrictively, thus ignoring a range of symptoms that are common yet, from the point of view of biomedicine, every bit as anomalous. On the other end are those who manage the category inclusively, including single, transient and relatively trivial symptoms. Importantly, however, the lack of consensus about the proper functioning of the junk drawer is silent, and also widely unrecognized (or ignored) by the scientists themselves. Although the category thus is a common interest and a ‘meeting ground’ for the scientists, it is not (yet) a site of interaction and exchange.

Though the claim that MUS are misfits in biomedical contexts is not new (Jutel, 2010: 230; Kirkengen et al., 2016: 496), the constitutive role of biomedical knowledge and research practice in medical science has, to my knowledge, not been explored and established empirically before.

### Assumed psychogenic aetiology as monster-adjustment

Medical scientists are not discarding or ‘monster-barring’ (Bloor, 1978) MUS, not treating the symptoms like waste. Instead, they are including them in the system of medical knowledge, subjecting them to research, using a containment device I call a junk drawer to protect the system’s orderly state from contamination. The MUS category thus serves this containment function. There is a sense of optimism to it, in that a junk drawer saves puzzles for later, allowing for future scientific understanding.

Regarding puzzle solving, historians of science and others suggest that when anomalous phenomena are not simply ignored or discarded, scientists will typically try somehow to make them fit in by making slight adjustments or creating new categories to the established order, or by proposing rule-exceptions that dampen or remove the anomalous character. These strategies are called ‘monster-adjustment’ (Bloor, 1978).

There are strong indications of a move towards such strategies in the sample, in the form of assumptions that unexplained physical symptoms are really symptoms of mental distress (e.g. Jutel, 2010). Such assumptions are criticized in the sample (e.g. **5-3**) but not nearly as often as they are taken for granted. They come to the fore, for instance, in the conviction that psychiatric therapy is appropriate for but hampered by uncooperative patients (e.g. **6-5**; **11-7**) or in the fact that only two articles in the sample actually investigate new hypotheses that MUS have somatic causes (**7-9**; **8-5**). As indicated in the analysis, the connection with somatization is one way this assumption seeps through. Other ways are through concepts that carry similar psychogenic assumptions – such as ‘alexithymia’ (**2-3**), ‘illness perceptions’ (**9-10**), and ‘somatovisceral illusions’ (**1-16**), each indicating that the patients have misunderstood the nature of their symptoms. As such, psychogenic assumptions are part of the biomedical doxa and the commitment to make MUS ‘fit in’. They may be interpreted as a form of monster-adjustment, pushing for the resolution of the anomalous character of present symptoms without signs.

This interpretation of MUS as caused by a misunderstanding on the patient’s part has been criticized by some social scientists as a form of blame-shifting (e.g. Horton-Salway, 2002; Jutel, 2010). Others have been more cautious. Greco (2012: 2365) warns against any knee-jerk criticism of psychogenic assumptions by social scientists. She argues that the medical profession might be right to treat MUS as psychogenic and that the question must be settled empirically. Inasmuch as ‘right’ indicates that it could work, I agree. However, from what evidence there is, it does not seem to do the trick: An important context where psychogenic assumptions are expressed is when researchers complain that patients reject psychiatric treatment (e.g. **6-5**; **11-7**). Undeterred, researchers have experimented with the reframing of psychiatric treatment as somatic treatment, trying to get patients into disguised psychiatric treatment (e.g. **8-8**, 2013: 300). In one study, this strategy is unashamedly presented as ‘a Trojan horse’ (**6-6**, 2011: 3) – without considering the risk that patients will learn to fear the GPs when they come bearing ‘therapeutic gifts’.

Somatization in its varieties has yet to succeed as a monster-adjusting strategy. Moreover, due to recent changes whereby all SD diagnoses have been ejected from formal classifications (American Psychiatric Association, 2013), the strategy might have to change. But that is the beauty of a ‘junk drawer category’: They can try again later.

### Standards and standardization

Members of the research community have lamented the lack of formalized and widely shared standards for classifying MUS. In the absence of standards, creative but highly varied classification practices characterize research. But what difference would shared standards make?

Formalizing a set of criteria, for instance relating to symptom count, impact and persistence, or the frequency of attendance, would probably enable researchers to study a more homogenous patient group than current practices allow for. However, standardized criteria do not necessarily make classification homogenous, as they must nevertheless be interpreted and applied in the course of situated practice (Bloor, 1997; Timmermans and Berg, 2003). This leaves room for substantial variation. In cases of MUS, standard criteria will not change the fact that counting or estimating the impact of symptoms is difficult work, with no definitive answers (Berg, 1992; Rosendal et al., 2013). Moreover, there are reasons to believe that what I call the core criterion is itself a major source of variation: studies indicate that even when criteria are formalized and shared, doctors disagree about where to draw the line between the explained and unexplained (e.g. Creed and Barsky, 2004: 404) – not least because they also disagree about the distinction between diseases and non-diseases (Smith, 2002; Tikkinen et al., 2012). This indicates a less than clear-cut line between the explained and the unexplained.

Standardizing the classification of MUS would therefore require a more thorough reflection over basic concepts such as disease, objective evidence and medical explanation. The potential advantage of doing so would be the ability to determine the value of MUS as a medical category – to test whether it is sensible and helpful to group patients based primarily on their lack of fit with the biomedical paradigm.

## Appendix

### Articles forming the sample

1-1: Nimnuan C, Hotopf M and Wessely S (2001) Medically unexplained symptoms: An epidemiological study in seven specialities. *Journal of Psychosomatic Research* 51(1): 361–367.
1-2: De Gucht V and Heiser W (2003) Alexithymia and somatisation: A quantitative review of the literature. *Journal of Psychosomatic Research* 54(5): 425–434.
1-3: Nimnuan C, Rabe-Hesketh S, Hotopf M, et al. (2001) How many functional somatic syndromes? *Journal of Psychosomatic Research* 51(4): 549–557.
1-4: Fink P and Schröder A (2010) One single diagnosis, bodily distress syndrome, succeeded to capture 10 diagnostic categories of functional somatic syndromes and somatoform disorders. *Journal of Psychosomatic Research* 68(5): 415–426.
1-5: Kroenke K (2006) Physical symptom disorder: A simpler diagnostic category for somatization-spectrum conditions. *Journal of Psychosomatic Research* 60(4): 335–339.
1-6: Frostholm L, Oernboel E, Christensen KS, et al. (2007) Do illness perceptions predict health outcomes in primary care patients? A 2-year follow-up study. *Journal of Psychosomatic Research* 62(2): 129–138.
1-7: Salmon P, Ring A, Dowrick CF, et al. (2005) What do general practice patients want when they present medically unexplained symptoms, and why do their doctors feel pressurized? *Journal of Psychosomatic Research* 59(4): 255–260.
1-8: Voigt K, Nagel A, Meyer B, et al. (2010) Towards positive diagnostic criteria: A systematic review of somatoform disorder diagnoses and suggestions for future classification. *Journal of Psychosomatic Research* 68(5): 403–414.
1-9: Jackson J, Fiddler M, Kapur N et al. (2006) Number of bodily symptoms predicts outcome more accurately than health anxiety in patients attending neurology, cardiology, and gastroenterology clinics. *Journal of Psychosomatic Research* 60(4): 357–363.
1-10: Creed FH, Davies I, Jackson J, et al. (2012) The epidemiology of multiple somatic symptoms. *Journal of Psychosomatic Research* 72(4): 311–317.
1-11: Witthöft M and Rubin GJ (2013) Are media warnings about the adverse health effects of modern life self-fulfilling? An experimental study on idiopathic environmental intolerance attributed to electromagnetic fields (IEI-EMF). *Journal of Psychosomatic Research* 74(3): 206–212.
1-12: van der Windt DA, Dunn KM, Spies-Dorgelo, MN, et al. (2008) Impact of physical symptoms on perceived health in the community. *Journal of Psychosomatic Research* 64(3): 265–274.
1-13: Leiknes KA, Finset A, Moum T, et al. (2006) Methodological issues concerning lifetime medically unexplained and medically explained symptoms of the Composite International Diagnostic Interview: A prospective 11-year follow-up study. *Journal of Psychosomatic Research* 61(2): 169–179.
1-14: Kolk AMM, Schagen S and Hanewald GJFP (2004) Multiple medically unexplained physical symptoms and health care utilization: Outcome of psychological intervention and patient-related predictors of change. *Journal of Psychosomatic Research* 57(4): 379–389.
1-15: Körber S, Frieser D, Steinbrecher N, et al. (2011) Classification characteristics of the Patient Health Questionnaire-15 for screening somatoform disorders in a primary care setting. *Journal of Psychosomatic Research* 71(3): 142-147.
1-16: Bogaerts K, Millen A, Li W, et al. (2008) High symptom reporters are less interoceptively accurate in a symptom-related context. *Journal of Psychosomatic Research* 65(5): 417–424.
1-17: Dimsdale JE, Creed F, Escobar J, et al. (2013) Somatic symptom disorder: an important change in DSM. *Journal of Psychosomatic Research* 75(3): 223–228.
1-18: Houtveen JH and Oei NY (2007) Recall bias in reporting medically unexplained symptoms comes from semantic memory. *Journal of Psychosomatic Research* 62(3): 277–282.
1-19: Kisely S and Simon G (2006) An international study comparing the effect of medically explained and unexplained somatic symptoms on psychosocial outcome. *Journal of Psychosomatic Research* 60(2): 125–130.
1-20: Brown RJ, Brunt N, Poliakoff E, et al. (2010) Illusory touch and tactile perception in somatoform dissociators. *Journal of Psychosomatic Research* 69(3): 241–248.
2-1: Kroenke K (2007) Efficacy of treatment for somatoform disorders: A review of randomized controlled trials. *Psychosomatic Medicine* 69(9): 881–888.
2-2: Kirmayer LJ and Sartorius N (2007) Cultural models and somatic syndromes. *Psychosomatic Medicine* 69(9): 832–840.
2-3: Mattila AK, Kronholm E, Jula A, et al. (2008) Alexithymia and somatization in general population. *Psychosomatic Medicine* 70(6): 716–722.
2-4: Sumathipala A (2007) What is the evidence for the efficacy of treatments for somatoform disorders? A critical review of previous intervention studies. *Psychosomatic Medicine* 69(9): 889–900.
2-5: Frostholm L, Fink P, Oernboel E, et al. (2005) The uncertain consultation and patient satisfaction: The impact of patients’ illness perceptions and a randomized controlled trial on the training of physicians’ communication skills. *Psychosomatic Medicine* 67(6): 897–905.
2-6: Rief W, Mewes R, Martin A, et al. (2010) Are psychological features useful in classifying patients with somatic symptoms? *Psychosomatic Medicine* 72(7): 648–655.
2-7: Bailer J, Witthöft M, Paul C, et al. (2005) Evidence for overlap between idiopathic environmental intolerance and somatoform disorders. *Psychosomatic Medicine* 67(6): 921–929.
2-8: Jackson JL and Kroenke K (2008) Prevalence, impact, and prognosis of multisomatoform disorder in primary care: A 5-year follow-up study. *Psychosomatic Medicine* 70(4): 430–434.
2-9: Smith RC, Gardiner JC, Lyles JS, et al. (2005) Exploration of DSM-IV criteria in primary care patients with medically unexplained symptoms. *Psychosomatic Medicine* 67(1): 123.
2-10: Epstein RM, Shields CG, Meldrum SC, et al. (2006) Physicians’ responses to patients’ medically unexplained symptoms. *Psychosomatic Medicine* 68(2): 269–276.
3-1: Dirkzwager AJ and Verhaak PF (2007) Patients with persistent medically unexplained symptoms in general practice: Characteristics and quality of care. *BMC Family Practice* 8(1): 33.
3-2: Olde Hartman TC, Hassink-Franke LJ, Lucassen PL, et al. (2009) Explanation and relations. How do general practitioners deal with patients with persistent medically unexplained symptoms: A focus group study. *BMC Family Practice* 10(1): 68.
3-3: Aiarzaguena JM, Gaminde I, Grandes G, et al. (2009) Somatisation in primary care: Experiences of primary care physicians involved in a training program and in a randomised controlled trial. *BMC Family Practice* 10(1): 73.
3-4: Dowrick C, Gask L, Hughes JG, et al. (2008) General practitioners’ views on reattribution for patients with medically unexplained symptoms: a questionnaire and qualitative study. *BMC Family Practice* 9(1): 46.
3-5: Dwamena FC, Lyles JS, Frankel RM, et al. (2009) In their own words: Qualitative study of high-utilising primary care patients with medically unexplained symptoms. *BMC Family Practice* 10(1): 67.
3-6: Smits FT, Brouwer HJ, Zwinderman AH, et al. (2013) Morbidity and doctor characteristics only partly explain the substantial healthcare expenditures of frequent attenders: A record linkage study between patient data and reimbursements data. *BMC Family Practice* 14(1): 138.
3-7: Harkness EF, Grant L, O’Brien SJ, et al. (2013) Using read codes to identify patients with irritable bowel syndrome in general practice: A database study. *BMC Family Practice* 14(1): 183.
3-8: Harkness EF, Harrington V, Hinder S, et al. (2013) GP perspectives of irritable bowel syndrome–an accepted illness, but management deviates from guidelines: A qualitative study. *BMC Family Practice* 14(1): 92.
3-9: Rask MT, Andersen RS, Bro F, et al. (2014) Towards a clinically useful diagnosis for mild-to-moderate conditions of medically unexplained symptoms in general practice: A mixed methods study. *BMC Family Practice* 15(1): 118.
3-10: Halvorsen PA, Edwards A, Aaraas IJ, et al. (2013) What professional activities do general practitioners find most meaningful? Cross sectional survey of Norwegian general practitioners. *BMC Family Practice* 14(1): 41.
4-1: Kroenke K, Spitzer RL, Williams JB, et al. (2010) The patient health questionnaire somatic, anxiety, and depressive symptom scales: A systematic review. *General Hospital Psychiatry* 32(4): 345–359.
4-2: Fiddler M, Jackson J, Kapur N, et al. (2004) Childhood adversity and frequent medical consultations. *General Hospital Psychiatry* 26(5): 367–377.
4-3: Mewes R, Rief W, Brähler E, et al. (2008) Lower decision threshold for doctor visits as a predictor of health care use in somatoform disorders and in the general population. *General Hospital Psychiatry* 30(4): 349–355.
4-4: Morriss R, Dowrick C, Salmon P, et al. (2006) Turning theory into practice: Rationale, feasibility and external validity of an exploratory randomized controlled trial of training family practitioners in reattribution to manage patients with medically unexplained symptoms (the MUST). *General Hospital Psychiatry* 28(4): 343–351.
4-5: Lyles JS, Hodges A, Collins C, et al. (2003) Using nurse practitioners to implement an intervention in primary care for high-utilizing patients with medically unexplained symptoms. *General Hospital Psychiatry* 25(2): 63–73.
4-6: Salmon P, Wissow L, Carroll J, et al. (2007) Doctors’ responses to patients with medically unexplained symptoms who seek emotional support: criticism or confrontation? *General Hospital Psychiatry* 29(5): 454–460.
4-7: Steinbrecher N and Hiller W (2011) Course and prediction of somatoform disorder and medically unexplained symptoms in primary care. *General Hospital Psychiatry* 33(4): 318–326.
4-8: Salmon P, Wissow L, Carroll J, et al. (2008) Doctors’ attachment style and their inclination to propose somatic interventions for medically unexplained symptoms. *General Hospital Psychiatry* 30(2): 104–111.
4-9: Frostholm L, Ørnbøl E, Hansen HS, et al. (2010) Which is more important for outcome: The physician’s or the patient’s understanding of a health problem? A 2-year follow-up study in primary care. *General Hospital Psychiatry* 32(1): 1–8.
4-10: Husain N, Chaudhry I, Afsar S, et al. (2004) Psychological distress among patients attending a general medical outpatient clinic in Pakistan. *General Hospital Psychiatry* 26(4): 277–281.
5-1: Wileman L, May C and Chew-Graham CA (2002) Medically unexplained symptoms and the problem of power in the primary care consultation: A qualitative study. *Family Practice* 19(2): 178–182.
5-2: Reid S, Whooley D, Crayford T, et al. (2001) Medically unexplained symptoms—GPs’ attitudes towards their cause and management. *Family Practice* 18(5): 519–523.
5-3: Verhaak PF, Meijer SA, Visser AP, et al. (2006) Persistent presentation of medically unexplained symptoms in general practice. *Family Practice* 23(4): 414–420.
5-4: Salmon P, Peters S, Rogers A, et al. (2007) Peering through the barriers in GPs’ explanations for declining to participate in research: The role of professional autonomy and the economy of time. *Family Practice* 24(3): 269–275.
5-5: Woivalin T, Krantz G, Mäntyranta T, et al. (2004) Medically unexplained symptoms: Perceptions of physicians in primary health care. *Family Practice* 21(2): 199–203.
5-6: Lam TP, Goldberg DP, Dowell AC, et al. (2012) Proposed new diagnoses of anxious depression and bodily stress syndrome in ICD-11-PHC: An international focus group study. *Family Practice* 30(1): 76–87.
5-7: Allegretti A, Borkan J, Reis S, et al. (2010) Paired interviews of shared experiences around chronic low back pain: Classic mismatch between patients and their doctors. *Family Practice* 27(6): 676–683.
5-8: McGorm K, Burton C, Weller D, et al. (2010) Patients repeatedly referred to secondary care with symptoms unexplained by organic disease: Prevalence, characteristics and referral pattern. *Family Practice* 27(5): 479–486.
5-9: Swanson LM, Hamilton JC and Feldman MD (2010) Physician-based estimates of medically unexplained symptoms: A comparison of four case definitions. *Family Practice* 27(5): 487–493.
5-10: Olde Hartman TC, Hassink-Franke LJA, Dowrick C, et al. (2008) Medically unexplained symptoms in family medicine: Defining a research agenda. Proceedings from WONCA 2007. *Family Practice* 25(4): 266–271.
6-1: Interian A, Allen LA, Gara MA, et al. (2006) Somatic complaints in primary care: Further examining the validity of the Patient Health Questionnaire (PHQ-15). *Psychosomatics* 47(5): 392–398.
6-2: Feder A, Olfson M, Gameroff M, et al. (2001) Medically unexplained symptoms in an urban general medicine practice. *Psychosomatics* 42(3): 261–268.
6-3: Steinbrecher N, Koerber S, Frieser D, et al. (2011) The prevalence of medically unexplained symptoms in primary care. *Psychosomatics* 52(3): 263–271.
6-4: Ciechanowski PS, Katon WJ, Russo JE, et al. (2002) Association of attachment style to lifetime medically unexplained symptoms in patients with hepatitis C. *Psychosomatics* 43(3): 206–212.
6-5: Martin A, Rauh E, Fichter M, et al. (2007) A one-session treatment for patients suffering from medically unexplained symptoms in primary care: A randomized clinical trial. *Psychosomatics* 48(4): 294–303.
6-6: Katsamanis M, Lehrer PM, Escobar JI, et al. (2011) Psychophysiologic treatment for patients with medically unexplained symptoms: A randomized controlled trial. *Psychosomatics* 52(3): 218–229.
6-7: Andersen NLT, Eplov LF, Andersen JT, et al. (2013) Health care use by patients with somatoform disorders: A register-based follow-up study. *Psychosomatics* 54(2): 132–141.
6-8: Van den Berg B, Yzermans CJ, Van der Velden PG, et al. (2009) Risk factors for unexplained symptoms after a disaster: A five-year longitudinal study in general practice. *Psychosomatics* 50(1): 69–77.
6-9: Ávila LA (2006) Somatization or psychosomatic symptoms? *Psychosomatics* 47(2): 163–166.
7-1: Salmon P, Dowrick CF, Ring A, et al. (2004) Voiced but unheard agendas: Qualitative analysis of the psychosocial cues that patients with unexplained symptoms present to general practitioners. *British Journal of General Practice* 54(500): 171–176.
7-2: Burton C (2003) Beyond somatisation: A review of the understanding and treatment of medically unexplained physical symptoms (MUPS). *British Journal of General Practice* (488): 231–239.
7-3: Dowrick CF, Ring A, Humphris GM, et al. (2004) Normalisation of unexplained symptoms by general practitioners: A functional typology. *British Journal of General Practice* 54(500): 165–170.
7-4: Olde Hartman TC, Lucassen PL, van de Lisdonk EH, et al. (2004) Chronic functional somatic symptoms: A single syndrome? *British Journal of General Practice* 54(509): 922–927.
7-5: Paterson C, Taylor RS, Griffiths P, et al. (2011) Acupuncture for ‘frequent attenders’ with medically unexplained symptoms: A randomised controlled trial (CACTUS study). *British Journal of General Practice* 61(587): e295–e305.
7-6: Rugg S, Paterson C, Britten N, et al. (2011) Traditional acupuncture for people with medically unexplained symptoms: A longitudinal qualitative study of patients’ experiences. *British Journal of General Practice* 61(587): e306–e315.
7-7: Cape J, Geyer C, Barker C et al. (2010) Facilitating understanding of mental health problems in GP consultations: A qualitative study using taped-assisted recall. *British Journal of General Practice* 60(580): 837–845.
7-8: Budtz-Lilly A, Vestergaard M, Fink P, et al. (2015) Patient characteristics and frequency of bodily distress syndrome in primary care: A cross-sectional study. *British Journal of General Practice* 65(638): e617–e623.
7-9: Koch H, van Bokhoven MA, ter Riet G, et al. (2009) Ordering blood tests for patients with unexplained fatigue in general practice: What does it yield? Results of the VAMPIRE trial. *British Journal of General Practice* 59(561): e93–e100.
8-1: Van der Feltz-Cornelis CM, van Oppen P, Ad HJ, et al. (2006) Randomised controlled trial of a collaborative care model with psychiatric consultation for persistent medically unexplained symptoms in general practice. *Psychotherapy and Psychosomatics* 75(5): 282–289.
8-2: Sirri L, Fava GA and Sonino N (2013) The unifying concept of illness behavior. *Psychotherapy and Psychosomatics* 82(2): 74–81.
8-3: Smith RC, Korban E, Kanj M, et al. (2004) A method for rating charts to identify and classify patients with medically unexplained symptoms. *Psychotherapy and Psychosomatics* 73(1): 36–42.
8-4: Toft T, Rosendal M, Ørnbøl E, et al. (2010) Training general practitioners in the treatment of functional somatic symptoms: Effects on patient health in a cluster-randomised controlled trial (the Functional Illness in Primary Care study). *Psychotherapy and Psychosomatics* 79(4): 227–237.
8-5: Buffington CT (2009) Developmental influences on medically unexplained symptoms. *Psychotherapy and Psychosomatics* 78(3): 139–144.
8-6: Schaefert R, Kaufmann C, Wild B, et al. (2013) Specific collaborative group intervention for patients with medically unexplained symptoms in general practice: A cluster randomized controlled trial. *Psychotherapy and Psychosomatics* 82(2): 106–119.
8-7: Schweickhardt A, Larisch A, Wirsching M, et al. (2007) Short-term psychotherapeutic interventions for somatizing patients in the general hospital: A randomized controlled study. *Psychotherapy and Psychosomatics* 76(6): 339–346.
8-8: Van Ravesteijn H, Lucassen P, Bor H, et al. (2013) Mindfulness-based cognitive therapy for patients with medically unexplained symptoms: a randomized controlled trial. *Psychotherapy and Psychosomatics* 82(5): 299–310.
8-9: Gerger H, Hlavica M, Gaab J, et al. (2015) Does it matter who provides psychological interventions for medically unexplained symptoms? A meta-analysis. *Psychotherapy and Psychosomatics* 84(4): 217–226.
9-1: Aiarzaguena JM, Grandes G, Gaminde I, et al. (2007) A randomized controlled clinical trial of a psychosocial and communication intervention carried out by GPs for patients with medically unexplained symptoms. *Psychological Medicine* 37(2): 283–294.
9-2: Sharpe M, Stone J, Hibberd C, et al. (2010) Neurology out-patients with symptoms unexplained by disease: illness beliefs and financial benefits predict 1-year outcome. *Psychological Medicine* 40(4): 689–698.
9-3: Spence M, Moss-Morris R and Chalder T (2005) The Behavioural Responses to Illness Questionnaire (BRIQ): a new predictive measure of medically unexplained symptoms following acute infection. *Psychological Medicine* 35(4): 583–593.
9-4: Reid S, Crayford T, Patel A, et al. (2003) Frequent attenders in secondary care: A 3-year follow-up study of patients with medically unexplained symptoms. *Psychological Medicine* 33(3): 519–524.
9-5: Burton C, McGorm K, Weller D, et al. (2011) Depression and anxiety in patients repeatedly referred to secondary care with medically unexplained symptoms: A case-control study. *Psychological Medicine* 41(3): 555–563.
9-6: Van den Berg B, Grievink L, van der Velden PG, et al. (2008) Risk factors for physical symptoms after a disaster: A longitudinal study. *Psychological Medicine* 38(4): 499–510.
9-7: Morriss R, Gask L, Dowrick C, et al. (2010) Randomized trial of reattribution on psychosocial talk between doctors and patients with medically unexplained symptoms. *Psychological Medicine* 40(2): 325–333.
9-8: Everitt B, Ismail K, David AS, et al. (2002) Searching for a Gulf War syndrome using cluster analysis. *Psychological Medicine* 32(8): 1371–1378.
9-9: Taylor RE, Marshall T, Mann A, et al. (2012) Insecure attachment and frequent attendance in primary care: a longitudinal cohort study of medically unexplained symptom presentations in ten UK general practices. *Psychological Medicine* 42(4): 855–864.
9-10: Frostholm L, Petrie KJ, Ørnbøl E, et al. (2014) Are illness perceptions related to future healthcare expenditure in patients with somatoform disorders? *Psychological Medicine* 44(13): 2903–2911.
10-1: Salmon P (2007) Conflict, collusion or collaboration in consultations about medically unexplained symptoms: The need for a curriculum of medical explanation. *Patient Education and Counseling* 67(3): 246–254.
10-2: Nettleton S, Watt I, O’Malley L, et al. (2005) Understanding the narratives of people who live with medically unexplained illness. *Patient Education and Counseling* 56(2): 205–210.
10-3: Salmon P and Young B (2005) Core assumptions and research opportunities in clinical communication. *Patient Education and Counseling* 58(3): 225–234.
10-4: Burbaum C, Stresing AM, Fritzsche K, et al. (2010) Medically unexplained symptoms as a threat to patients’ identity? A conversation analysis of patients’ reactions to psychosomatic attributions. *Patient Education and Counseling* 79(2): 207–217.
10-5: Olde Hartman TC, van Rijswijk E, van Dulmen S, et al. (2013) How patients and family physicians communicate about persistent medically unexplained symptoms. A qualitative study of video-recorded consultations. *Patient Education and Counseling* 90(3): 354–360.
10-6: Goudsmit EM, Ho-Yen DO and Dancey CP (2009) Learning to cope with chronic illness. Efficacy of a multi-component treatment for people with chronic fatigue syndrome. *Patient Education and Counseling* 77(2): 231–236.
10-7: Shattock L, Williamson H, Caldwell K, et al. (2013) ‘They’ve just got symptoms without science’: Medical trainees’ acquisition of negative attitudes towards patients with medically unexplained symptoms. *Patient Education and Counseling* 91(2): 249–254.
10-8: Monzoni CM, Duncan R, Grünewald R, et al. (2011) Are there interactional reasons why doctors may find it hard to tell patients that their physical symptoms may have emotional causes? A conversation analytic study in neurology outpatients. *Patient Education and Counseling* 85(3): e189–e200.
10-9: Aiarzaguena JM, Gaminde I, Clemente I, et al. (2013) Explaining medically unexplained symptoms: Somatizing patients’ responses in primary care. *Patient Education and Counseling* 93(1): 63–72.
10-10: Hagiwara N, Kashy DA and Penner LA (2014) A novel analytical strategy for patient–physician communication research: The one-with-many design. *Patient Education and Counseling* 95(3): 325–331.

## Notes

1. The search phrase was ‘TS=(‘medically unexplained symptoms’)’ (conducted January 7 2019). The increase is sizable also when weighted against the general increase in publications in English in the field of medicine (‘SU=medicine’), with 15% published in the first 10 years compared to 51% in the last ten.

2. In Norwegian, the word for junk drawer is ‘roteskuff’, which can be roughly translated as ‘messy drawer’. Although it refers to the same type of category, the Norwegian version more benignly hints at disorderliness without the connotations of ‘junk’ (waste, trash, worthless, etc.).

3. Bloor (1978: 255) also describes ‘exception-barring’, referring to the making of exceptions to a rule: Exception-barring leaves the validity of the rule intact, but limits ‘the span of its authority’.

4. The search phrase was ‘TS=(‘medically unexplained symptoms’)’. Lemmatization rules ensured that both ‘symptom’ and ‘symptoms’ were included. See https://images.webofknowledge.com/images/help/WOK/hs_topic.html, accessed January 26 2017. I note that Web of Science includes Medline in its database.

5. FSS should not be confused with functional somatic *symptoms* (Mayou and Farmer, 2002), a similar sounding but different concept and one that I have omitted from the analysis to avoid unnecessary confusion.

6. Some articles do not operationalize MUS because they are not explicitly researched. Instead, focus is on single complaints such as ‘chronic lower back pain’ (**3-7**; **5-7**), ‘idiopathic environmental intolerance’ (**2-7**), sometimes ‘attributed to electromagnetic fields’ (**1-11**) or ‘pseudoneurological symptoms’ (**1-12**). By connecting these conditions with MUS, the researchers manage to make their studies about something larger, but they also contribute to making the content of the category manifold and ambiguous.

7. The instrument is the Patient Health Questionnaire or PHQ-15, a checklist of 15 somatic symptoms for which ‘tissue abnormality’ is rarely found.

8. We may note that there is a contradiction in demanding a number of unexplained symptoms to determine *cases* of unexplained symptoms. The contradiction vanishes if we talk about category X and membership as predicated on a minimum number of phenomena Y: X and Y are thus homonyms, but not synonyms.

9. They used two screening instruments – Screening for Somatoform Symptoms or SOMS2 and the General Health Questionnaire or GHQ-12 – however, the threshold for inclusion was very low, excluding only patients who were are in perfect mental health (never having problems focusing, never losing sleep due to worry, always able to enjoy day-to-day activities, and so on).

10. Some opt instead to exclude symptoms that are gender-specific to avoid discriminating between male and female participants. For instance, one study excluded ‘menstrual symptoms’ due to the risk of a gender-skewed sample (see **5-8**, 2010: 481).

11. Other classifications include ‘bodily distress syndrome’ (**1-4**; **7-8**), ‘physical symptom disorder’ (**1-5**), ‘multisomatoform disorder’ (**2-8**), ‘somatic symptom disorder’ (**1-17**) and ‘the patient health questionnaire’ (**4-1**; **6-1**).
